# A new generation of direct X-ray detectors for medical and synchrotron imaging applications

**DOI:** 10.1038/s41598-020-76647-5

**Published:** 2020-11-18

**Authors:** A. Datta, Z. Zhong, S. Motakef

**Affiliations:** 1 CapeSym, Inc., Natick, MA 01760 USA; 2 National Synchrotron Light Source I & II, Brookhaven National Laboratory, Upton, NY 11973 USA

**Keywords:** Materials for devices, Applied physics, Imaging

## Abstract

Large-area X-ray imaging is one of the most widely used imaging modalities that spans several scientific and technological fields. Currently, the direct X-ray conversion materials that are being commercially used for large-area (> 8 cm × 4 cm without tiling) flat panel applications, such as amorphous selenium (a-Se), have usable sensitivities of up to only 30 keV. Although there have been many promising candidates (such as polycrystalline HgI_2_ and CdTe), none of the semiconductors were able to assuage the requirement for high energy (> 40 keV) large-area X-ray imaging applications due to inadequate cost, manufacturability, and long-term performance metrics. In this study, we successfully demonstrate the potential of the hybrid Methylammonium lead iodide (MAPbI_3_) perovskite-based semiconductor detectors in satisfying all the requirements for its successful commercialization in synchrotron and medical imaging. This new generation of hybrid detectors demonstrates low dark current under electric fields needed for high sensitivity X-ray imaging applications. The detectors have a linear response to X-ray energy and applied bias, no polarization effects at a moderate bias, and signal stability over long usage durations. Also, these detectors have demonstrated a stable detection response under BNL’s National Synchrotron Light Source II (NSLS-II) 70 keV monochromatic synchrotron beamline.

## Introduction

X-ray based imaging techniques are widely used in a wide range of applications. In particular, flat panel X-ray imagers (FPXIs) are now widely used in digital radiography, fluoroscopy, digital tomosynthesis, image-guided radiation therapy, and cone beam computed tomography, and applications in non-medical fields such as non-destructive, cultural heritage investigations, metrology, materials science research, geophysics, and homeland security^1–22^. In recent days, for the diagnosis of COVID-19, FPXIs are widely used for radiographic chest scans^23,24^. In this paper, we will discuss the prospective applications of FPXIs, based on hybrid Methylammonium lead iodide (CH_3_NH_3_PbI_3_, or MAPbI_3_) perovskite semiconductor, for medical X-ray radiography and synchrotron imaging.

**Figure 1:**
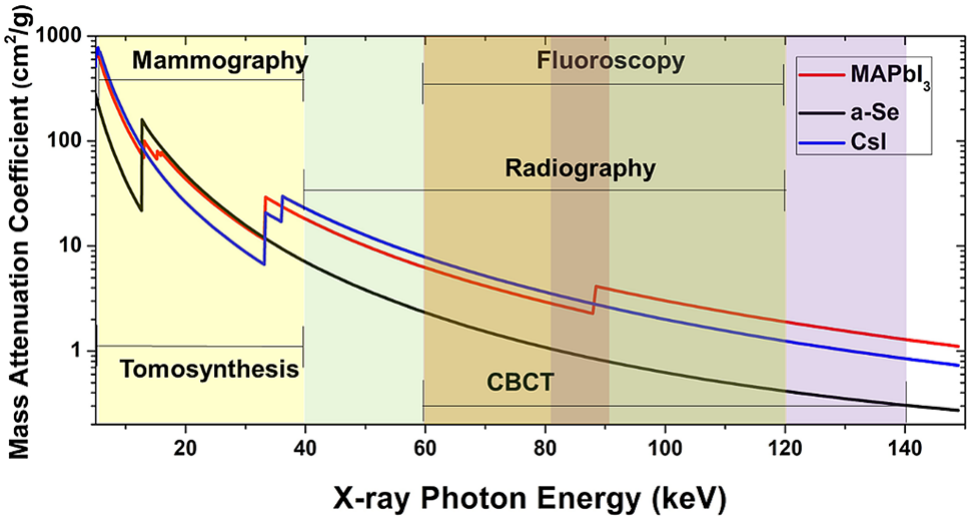
Mass attenuation coefficient versus X-ray energy for direct and indirect FPXI sensors, and MAPbI_3_ (calculations performed using NIST database). CBCT refers to cone beam CT.

**Figure 2:**
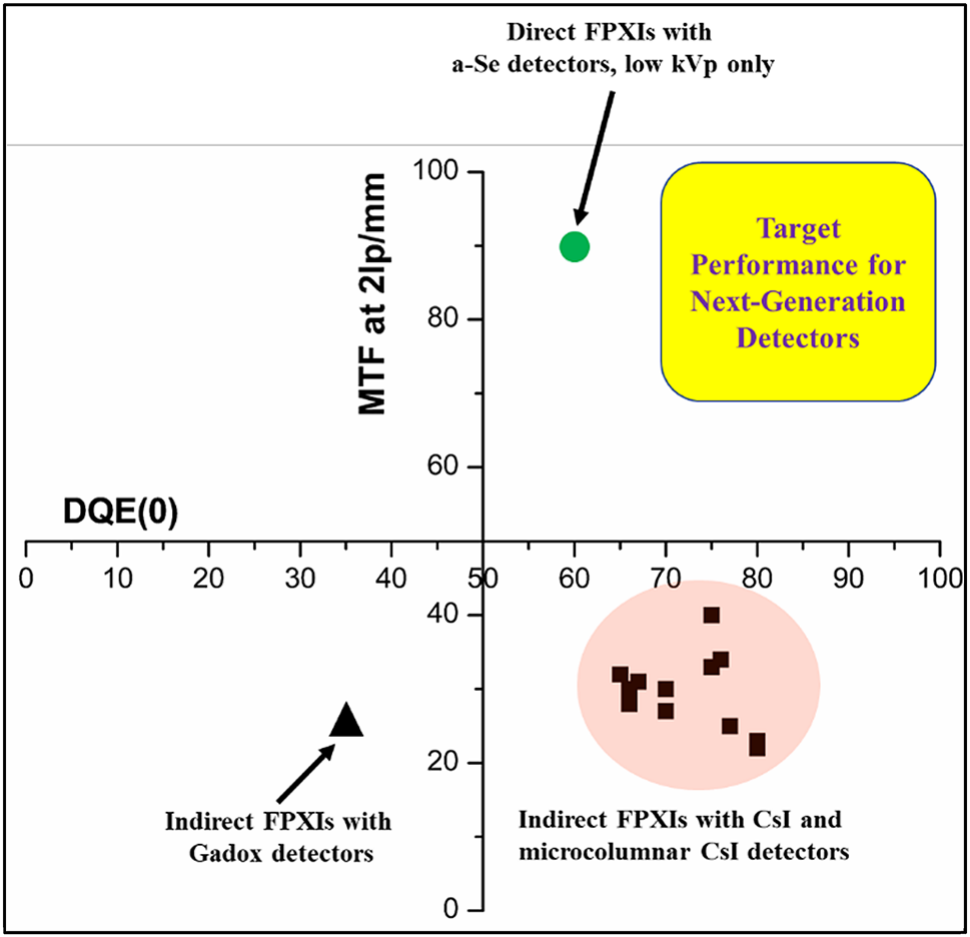
DQE versus MTF plot of commercial direct and indirect FPXIs. The target region for the FPXIs to be developed under this study is also shown. All the data points are from 17 COTS products. The DQE(0) CsI and Gadox data is for 1 µGy dose.

In the medical arena, it is well recognized that more and more patients are subjected to a higher level of accumulated radiation exposure and a concomitant increase in cancer risk^25^. Indirect and direct X-ray detection principles are the mechanisms by which the X-rays absorbed in the detector are converted to an electrical signal for data processing. Indirect conversion detectors are based on scintillating films where the absorbed X-ray produces photons in the scintillator, and the photons are subsequently detected by a photosensor. Direct conversion detectors are based on semiconductors films where the absorbed X-rays produce electron and hole charge carriers in the detector, which then drift towards the device electrodes under an imposed bias. The most prominent indirect conversion detector materials are CsI and Gd_2_O_2_S. Amorphous selenium (a-Se) is the only direction conversion material used in commercial FPXIs. Indirect FPXIs with scintillating layers (such as microcolumnar CsI and Gd_2_O_2_S have high detective quantum efficiency (DQE) and are the detectors of choice for all hard X-ray imaging applications. However, their relatively low image contrast, higher quantum noise, X-ray scattering (low modulation transfer function (MTF), and cone beam artifacts do not allow for X-ray dose reduction beyond current clinical levels. Direct FPXIs, however, have a higher DQE, MTF, and higher contrast-to-noise (CNR) ratio that makes them suitable for imaging fine anatomic structures and, in principle, lowering of X-ray dosage relative to indirect FPXIs. Figure 1 shows the mass attenuation coefficient of CsI and a-Se versus X-ray energy used in indirect and direct FPXIs, respectively. At energies up to 40 keV, a-Se has a higher attenuation coefficient than CsI. Beyond 40 keV, however, the attenuation coefficient of a-Se is lower than that of CsI by close to one order of magnitude, rendering a-Se direct conversion FPXIs impractical for chest and torso imaging as well as in any type of tomography applications that operate at energies > 40 keV. Mammography is the biggest market for current direct FPXIs, yet these FPXIs lose their effectiveness for dense breast tissues. Figure 2 shows a plot of MTF versus DQE for direct and indirect commercial FPXIs. Clearly, the indirect FPXIs have a much smaller MTF than direct FPXIs based on a-Se. The highlighted region denotes our best estimate on the expected performance of next-generation X-ray detectors that combine the best properties of both indirect and direct FPXIs.

Materials engineering and discovery have significantly benefitted from synchrotron-based imaging techniques in understanding the interplay between atomic-scale structures and macroscopic properties. Modern X-ray imaging techniques, such as coherent X-ray diffraction imaging and phase-contrast imaging, require high beam coherence and highly penetrating X-rays. In the current third-generation synchrotrons, such coherence is only attainable for the lower end of the hard X-ray spectrum. The new generation of synchrotrons, however, achieves enhanced coherence at beam energies higher than 40 keV. This increase in coherence benefits a number of scientific research areas: (1) intensely focused and coherent X-ray beams will dramatically advance nanoscale imaging, nano-spectroscopy, and nano-diffraction; (2) coherent diffractive imaging and ptychography will reach spatial resolution approaching atomic length-scales and permit time-resolved studies; and (3) the statistical information derived from photon correlation spectroscopy will open up previously inaccessible timescales and length-scales^26^. These new prospects for non-destructive imaging require high spatial resolution and efficient detectors, which again can only be successfully fulfilled using high sensitivity FPXIs.

A typical three-dimensional lead halide perovskite structure consists of eight octahedra of lead halide with Pb at the center. When the halide ions are changed, the displacement of the octahedra directly adjusts the bandgap of this class of material. The size of the cation (such as CH_3_NH_3_^+^) is an important factor for the formation of the cubo-octahedral perovskite structure by coordination with the anions^27^. MAPbI_3_ has emerged as a new generation of photovoltaic materials that achieved high power conversion efficiencies of around 23% within four years of their development. It is also an excellent candidate for radiation detection due to the presence of high-Z elements Pb. MAPbI_3_ has a very high attenuation coefficient, especially at high energies, similar to HgI_2_. MAPbI_3_ can, however, be produced at much higher thicknesses than HgI_2_. The strong spin-orbital coupling of MAPbI_3_ perovskites due to the presence of heavy elements and inversion asymmetry in the crystal structure results in Rashba effects^28,29^. Due to this, MAPbI_3_ single crystals demonstrate high diffusion lengths of up to 175 µm^30^ and carrier lifetimes of 15 µs^31^. Moreover, the recombination of charge carriers also minimizes in perovskite single crystals and polycrystalline thin films. Studies of Rashba-type effects in perovskites are still in their early stages, and further understanding of the phenomena and impact on charge-carrier recombination and transport is needed^28^. In our study, the mobility-lifetime (µτ) product of holes in 200 µm-thick polycrystalline MAPbI_3_ measured using the Hecht relation was found to be of the order of 10^–4^ cm^2^/V, similar to 250 µm thick polycrystalline HgI_2_ sensor and three orders of magnitude better than polycrystalline CZT. The µτ product of a single-crystalline MAPbI_3_ device is of the order of 10^–2^ cm^2^/V^32^, comparable to the state-of-the-art CZT device. Also, perovskites have a very low density of defects and traps within the bandgap^33^.

Although there has been a lot of progress in recent years on the development of MAPbI_3_ based X-ray detectors [e.g.^34^], none of these studies have demonstrated the repeatability and reliability of these devices. In addition, most of these studies are performed using thin sensor layers that significantly limits the X-ray stopping power^35^ or have used detector configurations that are incompatible with the widely used large-area high spatial resolution flat panel imaging^36^. There also has not been any stability data, which shows that these detectors can work for a considerable duration of time under X-ray irradiation. Reliability and repeatability have been the main problem with other direct halide semiconductor materials (such as HgI_2_). These detectors only demonstrate its excellent properties (such as the low dark current) for a few days under electric bias and then becomes unstable. As a result of this, even after a few decades of research, these are not commercially available for FPXI applications. In the following sections, we will discuss the fabrication techniques and properties of the large-area MAPbI_3_-based X-ray detectors that are prime candidates for a new generation of direct FPXIs. The main target of this study was to develop X-ray detectors that can be reliably and reproducibly used for the applications mentioned above.

## Results and discussion

Along with the efficiency and sensitivity of the X-ray detectors, the dark current density is a fundamentally important factor for the proper functioning of the readout matrix on which the halide sensor layer is being deposited. Nearly all detectors based on MAPbI_3_ have been reported to have a very high leakage current due to the relatively lower MAPbI_3_ bandgap. In order to tackle this problem, we focused on minimizing the dark current of the MAPbI_3_-based detectors with repeatable results while maintaining high X-ray sensitivity. Table 1 lists the performance of the MAPbI_3_-based detector configurations. Table S1 schematically shows the detector configurations. Figure S1 shows the legend for the various layers that are included in each configuration. Two types of polymers (polymers A and B in Figure S1) were used to fabricate the charge transporting layers. MAPbI_3_ detectors with different sensor thicknesses were fabricated in this study. The thickness range varied from 200 to 1400 µm. An SEM image of a typical MAPbI_3_ layer is shown in Supplementary Figure S2. Figures S3 and S4 show the X-ray characterization setups used in this study. More details on these setups are given in “Methods” section. The characterization experiments were started using a MAPbI_3_-based sensor with no additional charge manipulating layers, i.e., the MAPbI_3_ layer was biased directly from both sides. In subsequent steps, the charge controlling layers were added between the MAPbI_3_ sensor and the electrical contacts. A total of 16 configurations were tested, and the most promising six configurations are shown in Table 1.

**Figure 3:**
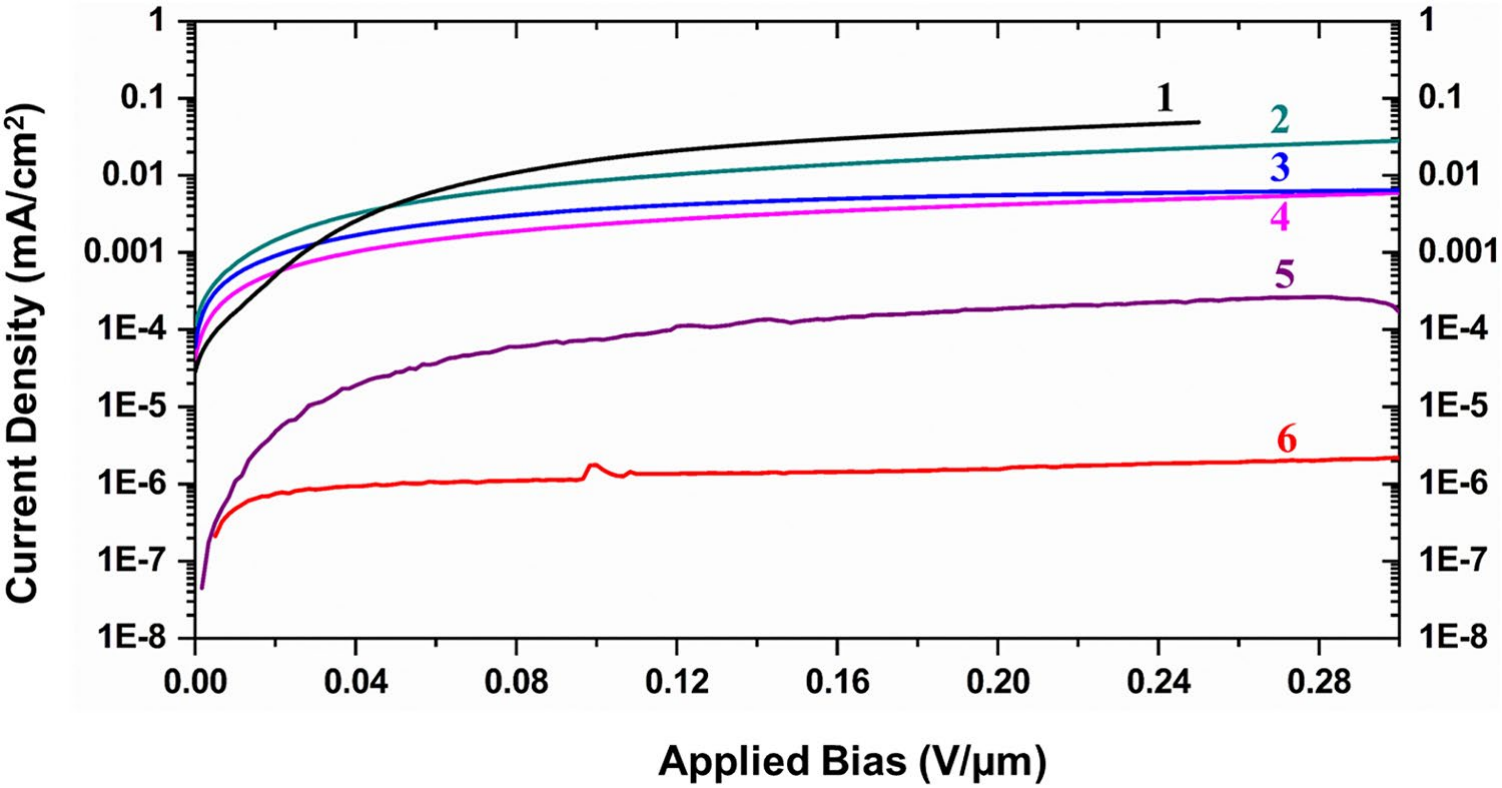
Current density versus voltage plot for detector configurations 1 through 6. See Table 1 for the details of the X-ray detector configurations.

The typical dark currents at an electric field of 0.08 V/µm for each configuration is also shown in the table. Figure 3 shows the current–voltage characteristics of the detectors for each configuration. As expected, a very high leakage current is seen in configuration 1 due to the lower MAPbI_3_ bandgap. The dark current baseline for configurations 1–3 was unstable and hence provided inferior detector stability. The lowest dark current was obtained using a single layer of polymer B between the MAPbI_3_ semiconductor layer and the contact (configuration 6). At 0.083 V/µm bias, the dark current was measured to be 1.29 × 10^–6^ mA/cm^2^. Five of these detectors were encapsulated using an optical epoxy and put under bias for *240 days*. The dark current baseline of the detectors was stable under constant bias voltage, and in fact, the dark current decreased to ~ 2.5 × 10^–7^ mA/cm^2^ over 240 days. The variations in the sensitivity of the MAPbI_3_ detector was less than ± 2%. X-ray response data for one of these detectors is shown in Supplementary Figure S5. On the other hand, after prolonged bias (~ 2 days), the baseline of all the other devices with configurations 4 and 5 started to show a significant amount of noise with up to a two-times increase in dark current. The encapsulation used for all these detectors were not fully optimized. Interaction with moisture and oxygen results in emanation of the organic species from the MAPbI_3_ matrix, leaving it Pb-rich, thereby deteriorating the photo-response of these detectors^34,37^. Hermetic encapsulation is essential for the long-term functioning of these detectors and is still a crucial challenge for perovskite materials that are currently under development for different applications^34,38^. Several cation and anion doping schemes have been suggested to alleviate this stability problem and can be used to stabilize these X-ray detectors for long-term applications under ambient atmospheres^39–41^. Future studies will be performed on optimizing such detectors for long-term X-ray detection applications.

**Table 1:** This table lists all the MAPbI_3_-based detector configurations that were tested in this study.

No	Device structure	Typical dark current at 0.08 V/µm (mA/cm^2^)	X-ray sensitivity at 0.08 V/µm (µC mGy^−1^ cm^−2^)
1	No charge limiting layer	1.1 × 10^–2^	17
2	Hole transporting and hole blocking layers	7 × 10^–3^	NA
3	Only hole blocking layer	3.1 × 10^–3^	NA
4	Only hole blocking layer	1.9 × 10^–3^	4.7
5	Only polymer A layer	2.3 × 10^–5^	5.8
6	Only ploymer B layer	1.5 × 10^–7^	6.1

The thickness of the electrical contacts was ~ 2 nm-thick, and the charge transport layers were ~ 2 µm-thick.

**Figure 4:**
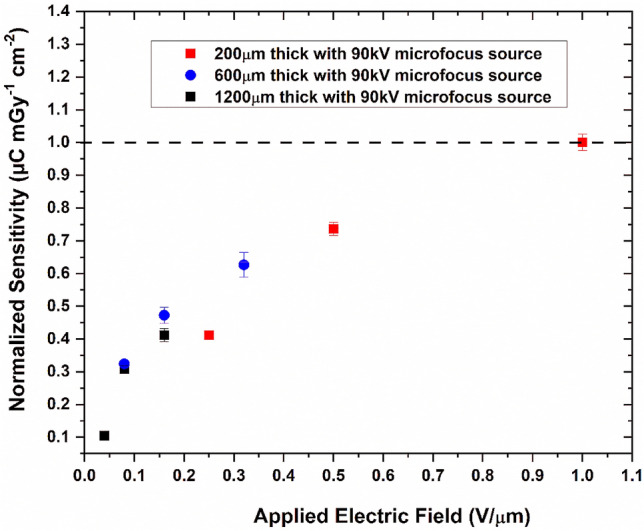
Normalized X-ray sensitivity of MAPbI_3_ detectors with different thicknesses as measured using the microfocus X-ray source set up. Notice the effects of trapping in the thicker detectors and the lower efficiency of the thinner detectors.

**Figure 5:**
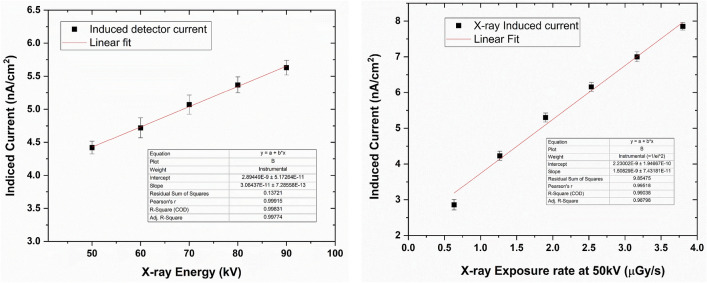
X-ray response of the 1200 µm-thick MAPbI_3_ detector biased at 0.042 V/µm. The highest exposure dose rate is 3.8 µGy/s.

**Figure 6:**
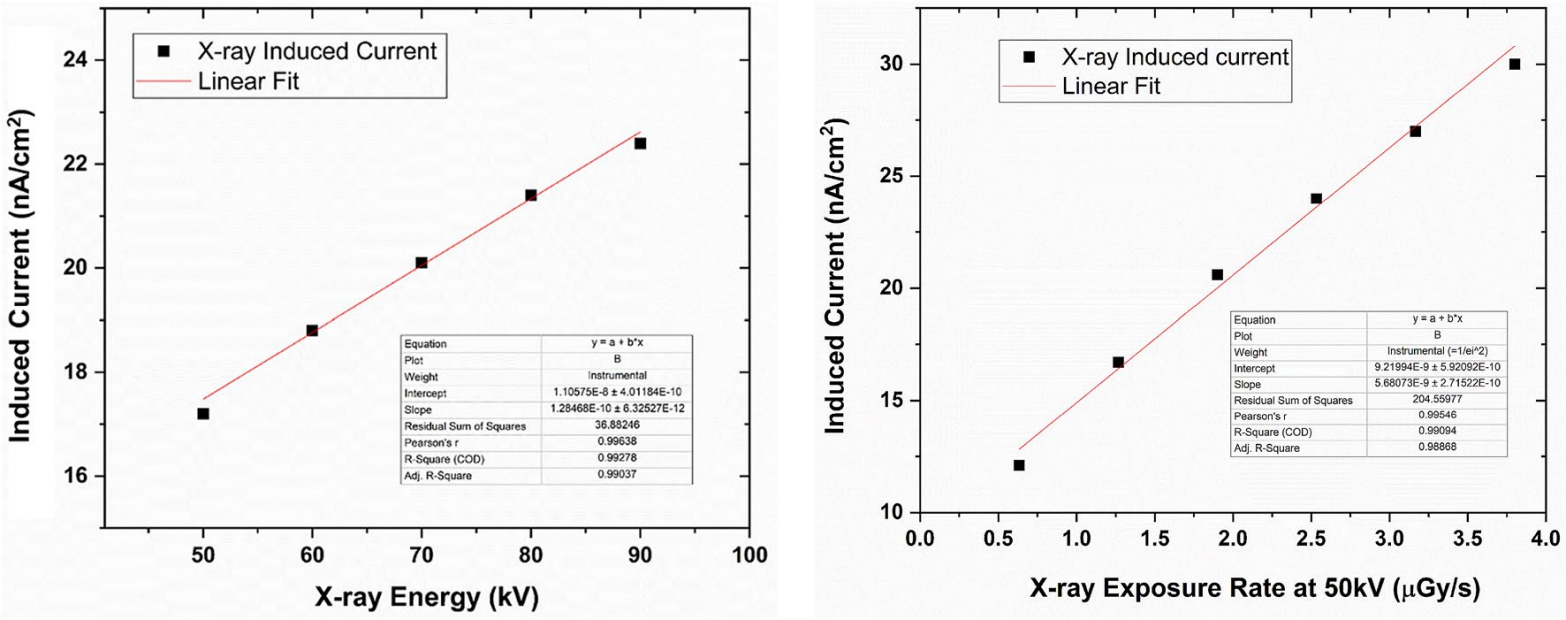
X-ray response of the 200 µm-thick MAPbI_3_ detector biased at 0.25 V/µm. The highest exposure dose rate is 3.8 µGy/s. The error values in the Y-axes are limited to 8.4 × 10^–11^ nA/cm^2^. The error bars in this data are not visible in the plot.

The X-ray sensitivity of the detectors was characterized using the set up shown in Figure S3. The detector-to-source distance was kept constant at around 20 cm. The detectors fabricated using configurations 1 through 5 demonstrated high sensitivities up to 17 µC mGy^−1^ cm^−2^ at an electric field of 0.08 V/µm. However, due to the lack of stability of the detectors and poor reproducibility, we focused our studies on configuration 6. The sensitivity values for the 1200 µm-thick detector were 1.9–7.5 µCmGy^−1^ cm^−2^ for 0.041–0.16 V/µm applied bias. For the 200 µm-thick detector, the sensitivity values were 7.5–13.5 µCmGy^−1^ cm^−2^ for 0.25–0.5 V/µm applied bias. Both these detectors were tested at 90 kV with 85 µA tube current. A comparison of the sensitivity values for detectors with different thicknesses are given in Fig. 4. Here we can see that the sensitivity increases as the applied bias is increased. Also, for similar electric fields, thinner detectors show higher sensitivity, showing the effects of charge trapping inside the thicker detectors. The plot also shows the lower efficiency of the 200 µm detector in comparison to the 600 and 1200 µm detectors. Figures 5 and 6 show the linearity with respect to the incoming X-ray energy and X-ray exposure rate of the 1200 µm- and 200 µm-thick detectors, respectively. As can be seen, the X-ray response of both these detectors are linear and hence shows the feasibility of linear X-ray response of MAPbI_3_-based detectors.

**Figure 7:**
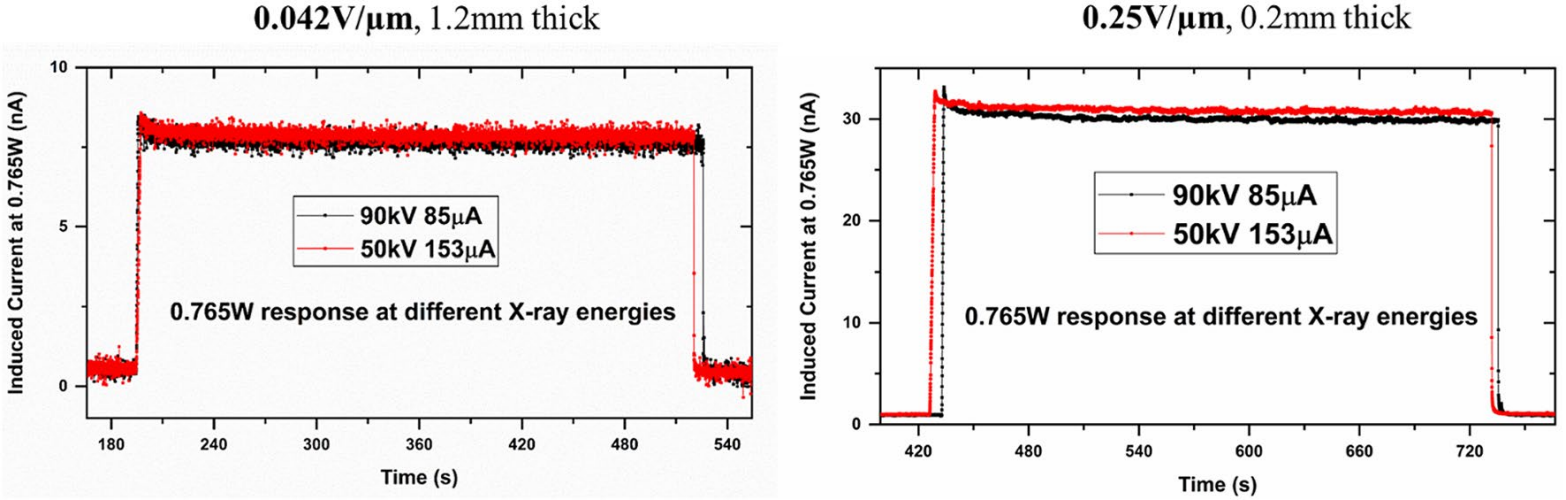
X-ray response of the 1200 µm- and 200 µm-thick MAPbI_3_ detector biased at 0.042 V/µm and 0.25 V/µm, respectively.

**Figure 8:**
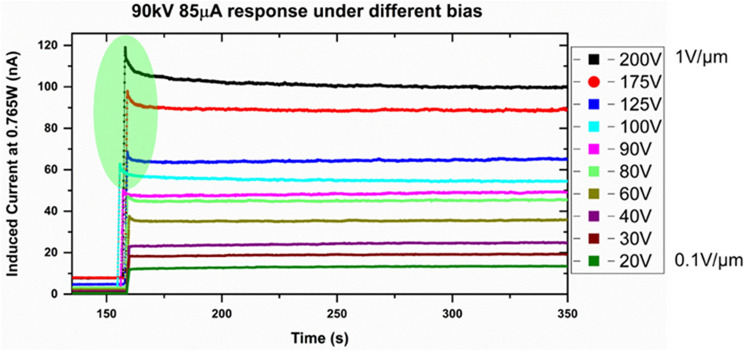
Polarization effect at the rising edge of the X-ray response in the 200 µm-thick MAPbI_3_ detector at higher bias voltages.

**Figure 9:**
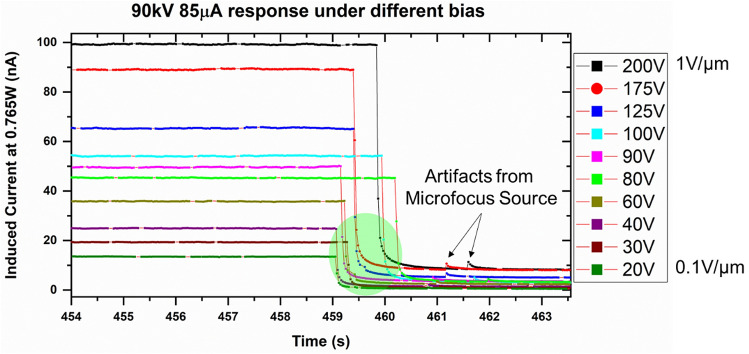
The falling edge of the X-ray response shows low decay lags in the 200 µm-thick MAPbI_3_ detectors at all voltages.

Figure 7 shows the response of the 1200 µm- and 200 µm-thick detectors at the same wattage. This again shows the highly linear and uniform response from two separate detectors. As expected, higher polarization effects were seen in the thicker detector. Figure 8 shows the difference in polarization of the detectors biased at different bias voltages. It is clear that higher bias voltages result in polarization effects, visible as a decay of the signal after the initial signal rise. The prolonged polarization effect turns off around 0.5 V/µm applied bias, whereas the fast polarization is eliminated at 0.2 V/µm. Figure 9 shows the falling edge of the 200 µm-thick MAPbI_3_ detector after turning off the X-ray tube. The charge trapping in bulk contributes to the decay lag in these detectors. The lower decay lag in these detectors as compared to other polycrystalline semiconductors such as a-Se is due to the presence of shallow defects in MAPbI_3_ crystallites^42^. Deeper traps result in longer decay times, thereby increasing the decay lag. Another factor that adds to this time lag is the delay in charge injection through the increased electric field generated due to the X-ray illumination at the contact electrodes and the barrier layer (such as the polymer B layer in configuration 6).

**Figure 10:**
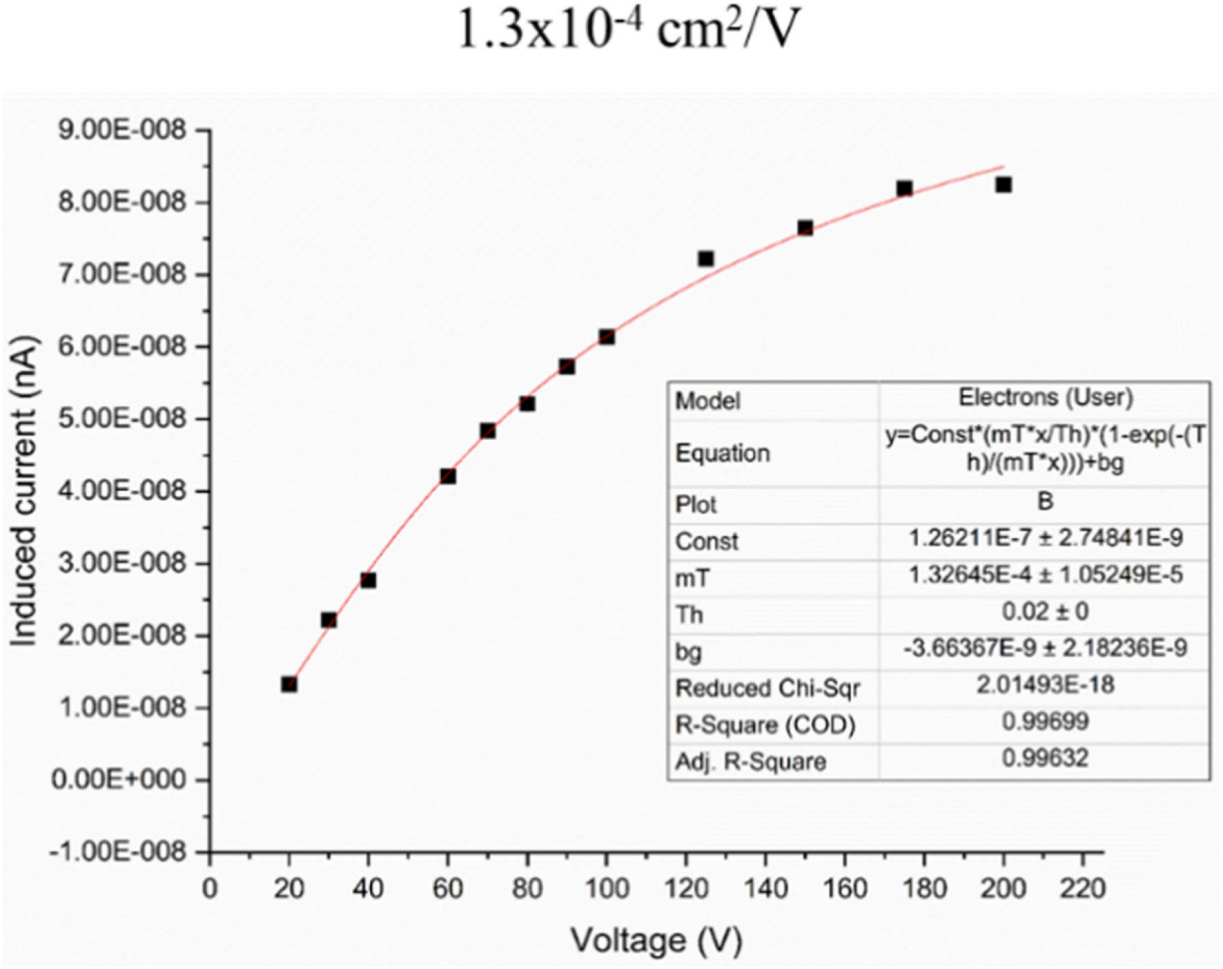
Mobility-lifetime calculations for detectors with 200 µm thickness.

We also estimated the mobility-lifetime characteristics of MAPbI_3_ detectors using the classical Hecht-equation and obtained mobility-lifetime values in the order of ~ 10^–4^ cm^2^/V. Figure 10 shows the data for one of these detectors. It is clear that these detectors possess excellent charge carrier properties and could potentially become the best performing sensor material for direct detection of higher energy X-rays.

**Figure 11:**
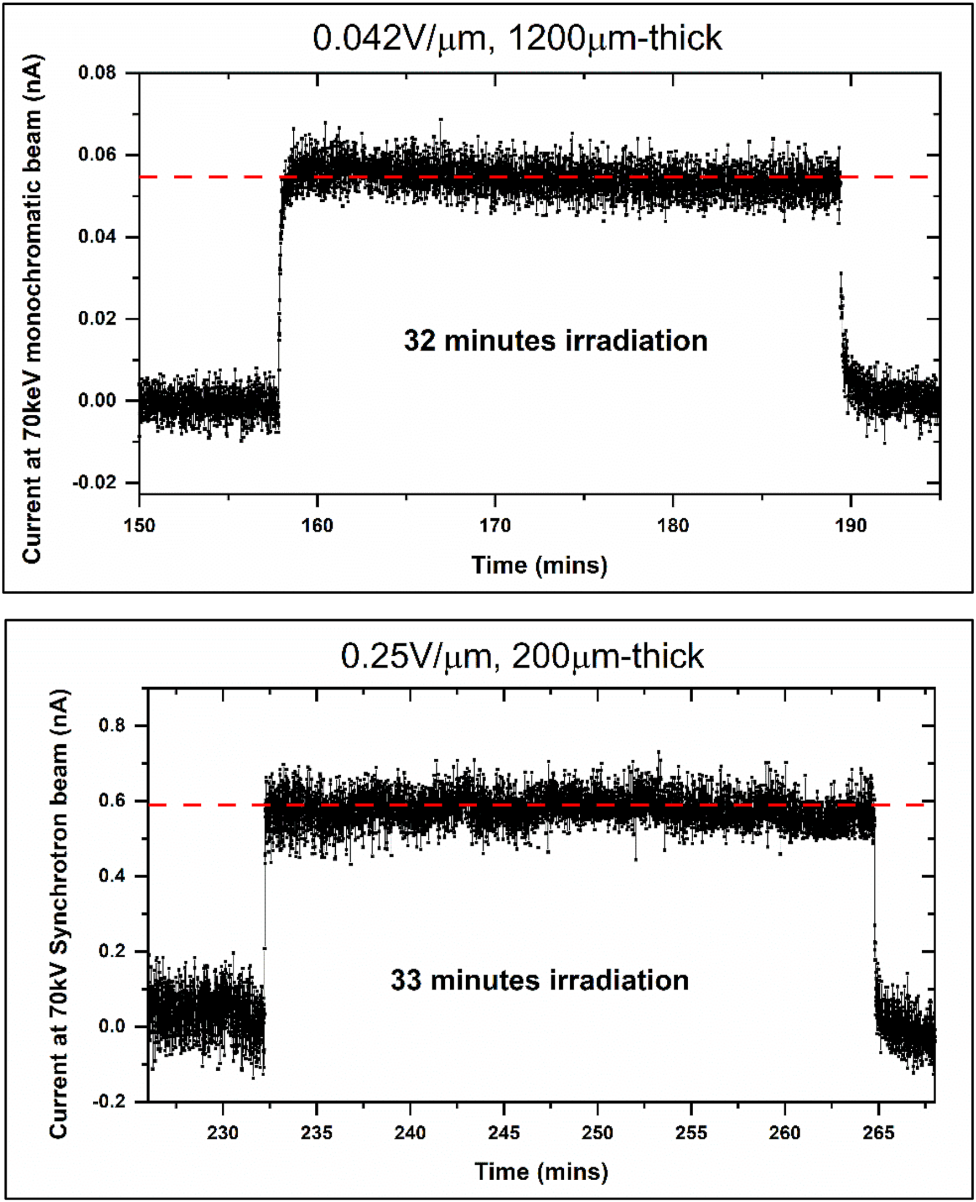
Monochromatic 70 keV synchrotron response of 1200 µm and 200 µm-thick MAPbI_3_ detectors biased at 50 V. The Y-axis is in nA and shows much lower current values as compared to microfocus X-ray responses due to the lower effective dose rates. The dashed straight lines show the average response of the detectors to the incoming monochromatic 70 keV synchrotron X-rays.

The performance of 1200 µm and 200 µm-thick detectors was also tested at the NSLS II XPD beamline at Brookhaven National Laboratory (BNL), Figure S4. The BNL beamline safety regulations restricted the allowed voltage for any new equipment (such as the X-ray detector) being operated in the beamline room, which in turn limited the maximum electric field to 0.25 V/µm. In addition, these detectors were not hermetically encapsulated and were exposed to an ambient atmosphere for three days before they were tested at the BNL NSLS-II beamline. Figure 11 shows the response of these detectors under a monochromatic 70 keV synchrotron X-ray for more than 30 min. Although polycrystalline MAPbI_3_ detectors yielded measurable responses under high energy monochromatic synchrotron radiation under a low applied bias, these responses merely represent the feasibility of these detectors for synchrotron detection and are not fully optimized. With further optimization, these detectors hold the potential to demonstrate much higher SNR, as demonstrated with the microfocus X-ray source. The future direction of this study will be focused on the development of large-area high spatial resolution FPXIs. This will include sensor material and detector structure optimization, design and fabrication of an appropriate pixelated backplane optimized for MAPbI_3,_ and testing of these detectors for synchrotron and medical imaging applications.

In conclusion, we established and validated the path forward for a new generation of polycrystalline X-ray detectors that have applications in numerous fields that require large area FPXIs, specifically in medical and synchrotron imaging. Not only MAPbI_3_-based semiconducting layers are highly efficient and highly sensitive to X-rays with sensitivity values as high as 13.5 µCmGy^−1^ cm^−2^ as measured using a microfocus X-ray source, but they are also easily manufacturable and highly reliable for long-term applications when optimally encapsulated. These multi-layered detectors exhibit extremely low dark currents in the range of ~ 1 nA/cm^2^ under a high bias voltage of 1 V/µm and ~ 150 pA/cm^2^ under a lower bias voltage of 0.25 V/µm, suitable for the fabrication of low noise FPXIs on active pixel array backplanes such as a-Si TFTs. We demonstrated a variety of performance and stability tests with these detectors, including detector response testing with monochromatic 70 keV synchrotron radiation at BNL. The epoxy-encapsulated detectors also showed stable dark current and X-ray detection sensitivity for over eight months in an ambient atmosphere.

## Methods

### Detector fabrication

All the physical MAPbI_3_ devices comprised of a 5 cm × 5 cm ITO-coated glass substrate (1850 Å thick ITO with a resistivity of 10–15 Ω per sq. in.) with Ag bus lines for electrical contact. We fabricated 2 cm × 2 cm MAPbI_3_ sensor layers that were 600 µm-thick and deposited 1 cm × 1 cm electrical contacts on the top. The polycrystalline MAPbI_3_ sensor layers were synthesized using a solvent-based process and deposited using doctor blading technique.

Specific molar ratios of CH_3_NH_3_I and lead iodide (99.999% trace metal basis) was added to a polar solvent such as *N*-Methyl-2-Pyrrolidone (NMP) or γ-butyrolactone typically at a ratio of 60:40 wt% (solvent to solute). It was observed that the grain characteristics of the MAPbI_3_ sensor are highly dependent on the starting stoichiometry of the MAI and PbI_2_ precursors. Supplementary Figure S6 shows the differences in the grain characteristics. This mixture was then placed in a convection oven at 100 °C and was vigorously mixed for 24 h. As observed, the reaction initiates within five minutes, and the colorless solution with white precursors turns dark grey. Once the solution appeared homogeneous with a smooth texture, a co-solvent (e.g. α-terpineol) was added to the mixture for thicker sensors. The solution was again placed inside an oven at 95 °C and was vigorously mixed for the next 24 h. The solution was decanted, and the viscous MAPbI_3_ was used to fabricate the X-ray detectors using the doctor blading technique. The hole-transporting and hole-blocking layers were fabricated by mixing MAPbI_3_ and MAPbBr_3_ with polymer A respectively. These layers were deposited using spin coating. A similar strategy was used by Kim et al. using polyimide based polymers^43^. The top and bottom electrodes were chosen to be chemically non-reactive oxide contacts (e.g., SnO_2_, ITO) for halide semiconductors, as shown in our previous study^44^.

### X-ray characterization

For the characterization of the X-ray performance of the detectors, we used the Thermo Scientific PXS5-928 digitally controlled 90 kV microfocus X-ray source. Figure S3 shows the X-ray set up used for determining the X-ray sensitivity of the detectors. It also shows a typical epoxy-encapsulated MAPbI_3_-based X-ray detector, which was tested using this set up as well as using the BNL NSLS II synchrotron beamline. Figure S4 shows the experimental set up at the BNL NSLS II beamline.

## Supplementary information

Supplementary Information.
