# Supplementary material for: A new generation of direct X-ray detectors for medical and synchrotron imaging applications
Source: Sci Rep. 2020 Nov 18;10:20097. doi: 10.1038/s41598-020-76647-5 (PMC7676260; doi:10.1038/s41598-020-76647-5)
Supplement: Supplementary file 1 — Supplementary Information. [file 41598_2020_76647_MOESM1_ESM.pdf]

A New Generation of Direct X-ray Detectors for Medical and Synchrotron Imaging Applications

A. Datta,[1] Z. Zhong,[2] and S. Motakef[1]

Supplementary Material

[1]CapeSym, Inc., 6 Huron Drive, Natick, MA 01760, US. Correspondence should be addressed to A.D. (email: datta@capesym.com)
[2]National Synchrotron Light Source I & II, Brookhaven National Laboratory, Upton, NY, 11973, USA

Table S1. This table lists the schematics of the $MAPbI_3$-based device structures that were tested in this study. The legend for the different layers are shown in Figure S1.

No.	Device structure	No.	Device structure
1	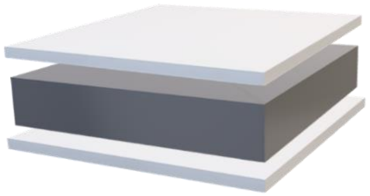	2	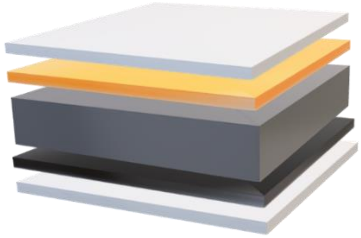
3	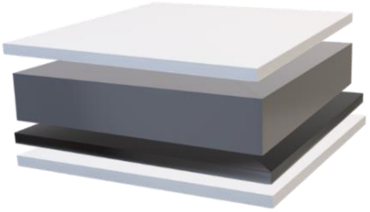	4	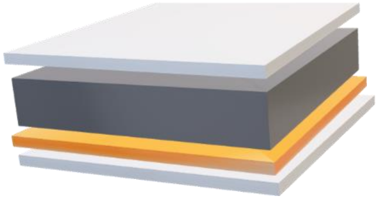
5	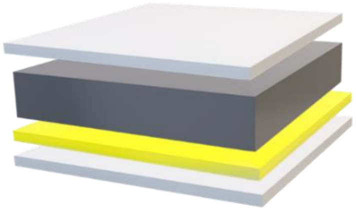	6	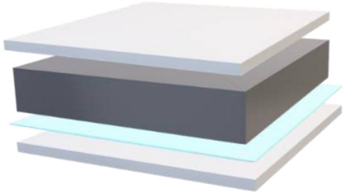

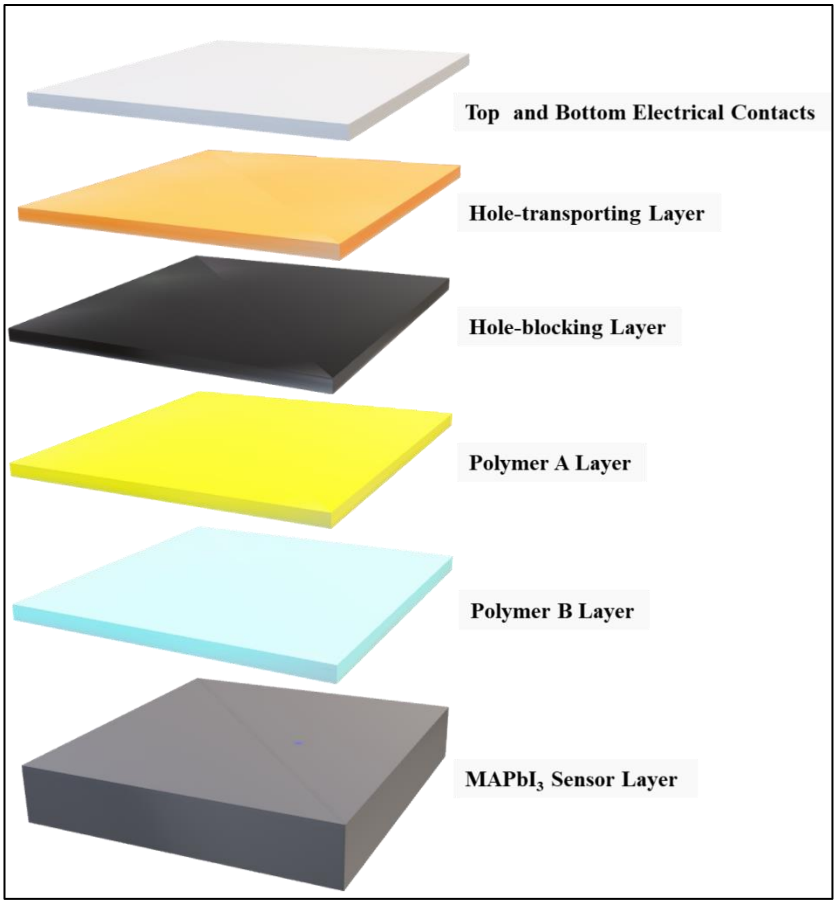

Figure S1. This figure shows the legend for Table I, demonstrating the various layers included in the various detector configurations.

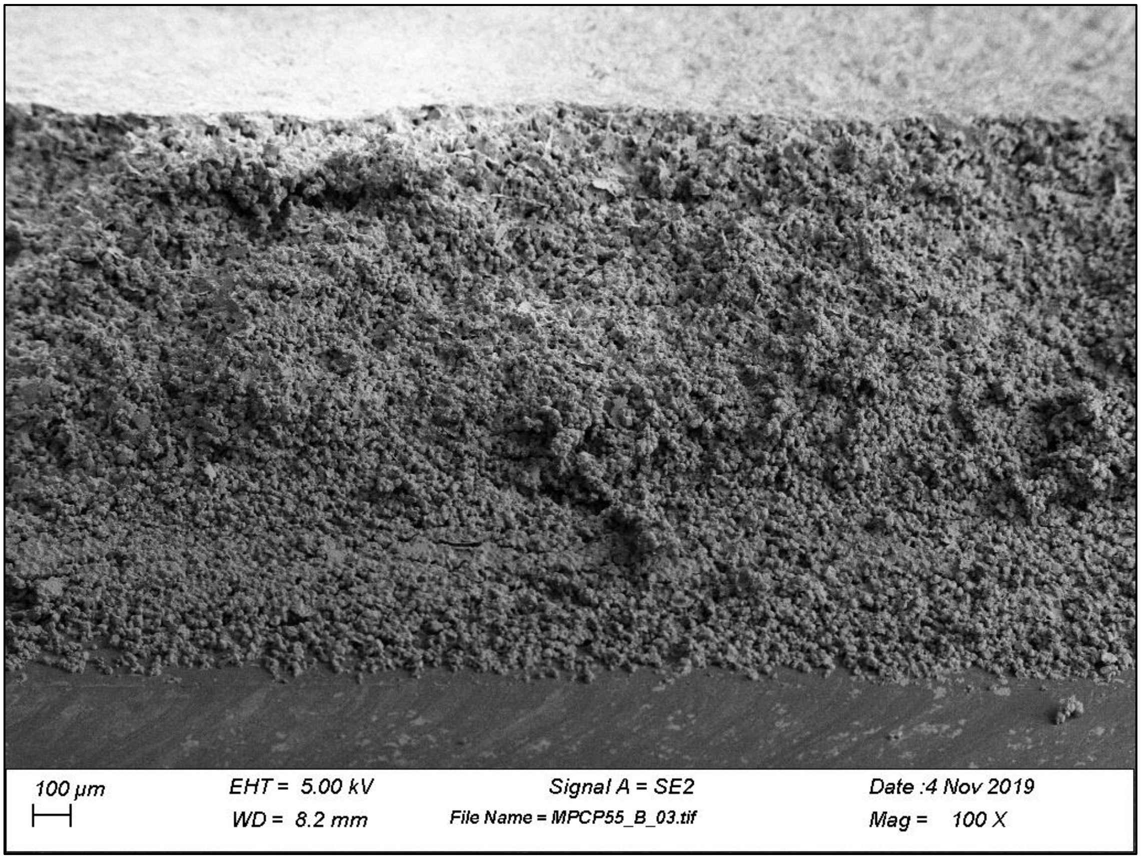

Figure S2. Side-view SEM image of a 1400µm-thick film fabricated using configuration 6.

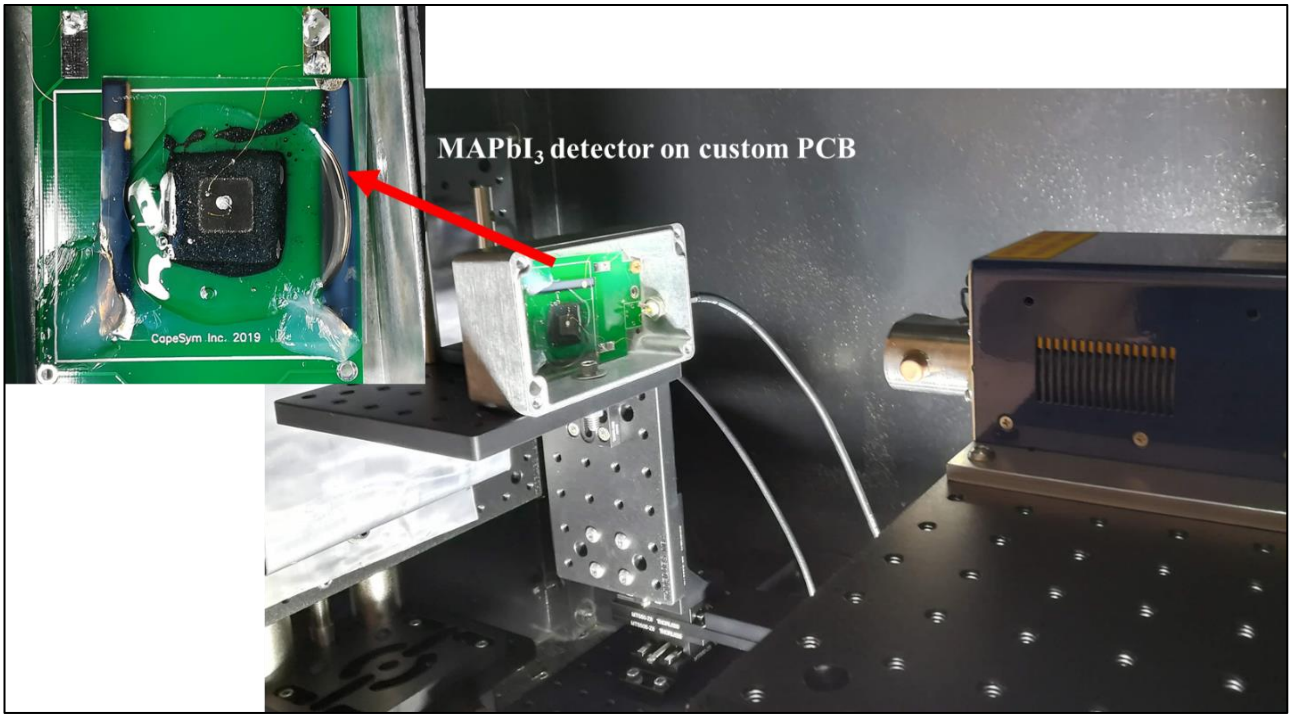

Figure S3. Microfocus X-ray characterization set up for the $MAPbI_3$ detectors. The detector was attached to a custom fabricated PCB. The same set of detectors were tested at the BNL NSLS II synchrotron beamline.

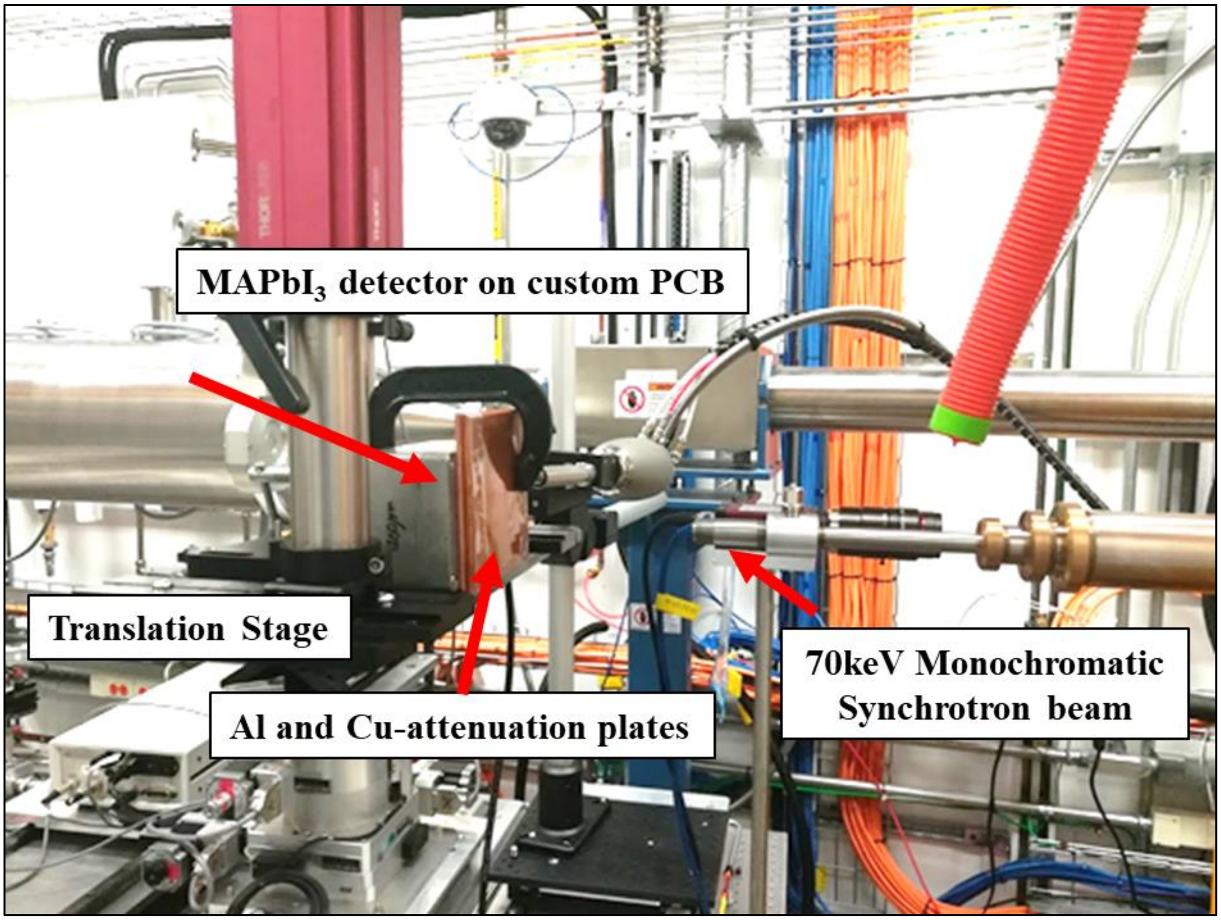

Figure S4. Experimental set-up for synchrotron response testing of $MAPbI_3$ detectors at the XPD beamline at the NSLS II beamline. The flux of the monochromatic 70keV beamline was set to 10^8 photons/mm^2-sec. 3.8mm-thick Al and 2mm-thick Cu plates were placed in front of the detector.

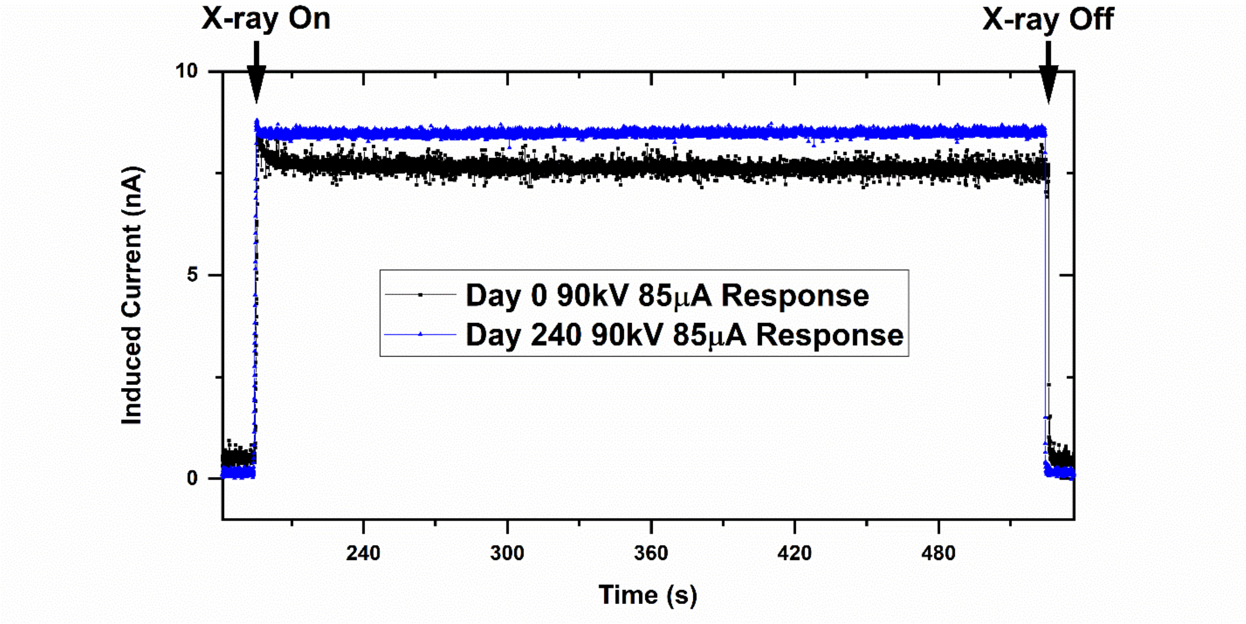

Figure S5. Stable X-ray response of a $MAPbI_3$ detector after storage in an ambient atmosphere for over eight months.

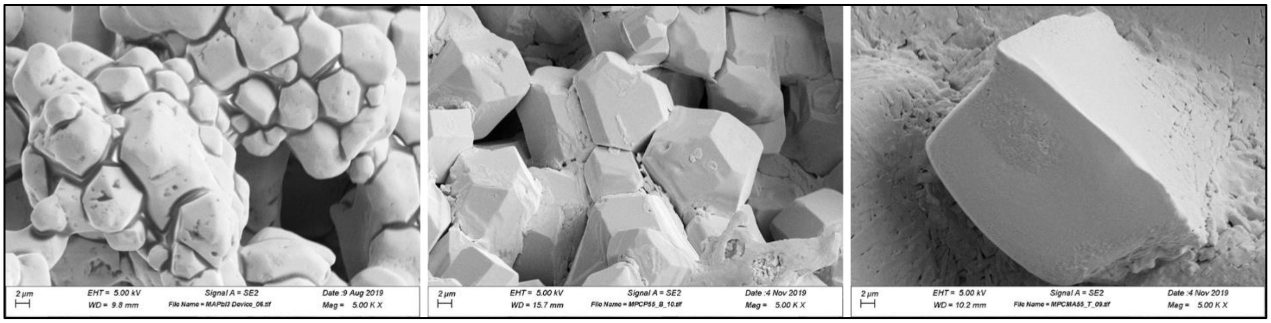

Figure S6. SEM images showing the differences in the grain shapes between films developed using three different initial stoichiometry: stoichiometric (left), Pb-rich (middle), and MA-rich (right). The scale shown in the description area below the SEM image represents 2μm.
